# Evaluation of the reliability and validity of the Early Childhood Oral Health Impact Scale (ECOHIS) questionnaire translated into Malagasy

**DOI:** 10.1186/s12955-020-01296-1

**Published:** 2020-02-24

**Authors:** Jeannot Randrianarivony, Justin Jacques Ravelomanantsoa, Noeline Razanamihaja

**Affiliations:** 1 IOSTM/University of Mahajanga, Mahajanga, Madagascar; 2 EDNES/University of Mahajanga, Mahajanga, Madagascar; 3 University Paris Diderot, Paris, France

**Keywords:** Preschool child, Oral health, Quality of life, ECOHIS, Validation

## Abstract

**Abstract:**

The Early Childhood Oral Health Impact Scale (ECOHIS) was developed to assess the impact of oral health conditions on the quality of life of preschool children and theirs families. The ECOHIS was originally developed in English language, translated and validated in several countries but no validated transcultural version of this questionnaire is currently available in Madagascar. The objectives of this cross-cultural study were to translate, validate and analyse the psychometric properties of the Malagasy version of ECOHIS.

**Method:**

The translation followed the forward-backward translation process. The Malagasy version obtained was pre-tested on ten mothers. Then, the questionnaire was administered by interview to 150 parents/guardians of children aged 3 to 5 years old frequenting two nursery schools in the town of Mahajanga to evaluate the reliability and validity of its psychometric characteristics. Reliability was assessed by the test-retest procedure with an interval of 15 days by using the intra-class correlation (ICC). Internal consistency was tested by Cronbach’s alpha coefficient. The validity of construct, discriminant and criterion were evaluated. Construct validity was evaluated by Spearman rank correlation and tested using exploratory factor analysis and partial confirmatory factor analysis. Discriminant validity was tested between groups of children presenting consequences of untreated decayed teeth. Clinical examination was performed using the decayed, missing, filled teeth (dmft) and the pulpal involvement, ulceration, fistula, abscess (pufa) indices for assessing dental caries and consequences of untreated decayed teeth.

**Results:**

The cultural adaptation showed that the respondents understood the questions. The intraclass correlation coefficient for test retest was 0.91. The internal consistency demonstrated a good reliability of the Malagasy-ECOHIS version with a Cronbach’s alpha coefficient of 0.88. The convergent validity evaluated by Pearson correlation coefficients provided positive and significant correlation values between all the items ranging from 0.26 to 0.72. Significant associations between the ECOHIS scores and the global rating of oral health supported the validity of the construct. Convergent and discriminant validity were obtained by testing the association of ECOHIS scores on the child and family sections with poor parental ratings of their child’s oral health, high caries experience and untreated decayed teeth consequences which were to be statistically significant.

**Conclusion:**

The results showed that this Malagasy version of ECOHIS questionnaire has shown good psychometric properties and could be used on Malagasy parents of preschool children to measure the impact of oral health status on the child and family quality of life.

## Introduction

In general, the oral health of individual is evaluated using quantitative clinical indicators such as the such as the decayed, missing, and filled permanent or temporary teeth index (DMFT/dmft) for dental caries and the community periodontal index (CPI/cpi) for periodontal diseases. These indicators measure the presence or the absence of diseases, and their severity and consequences, but not the daily impacts of different oral conditions on an individual’s life [1]. In recent decades, new methods of oral health qualitative measures have been developed to evaluate the physical, psychological, and social impact of oral conditions of individuals in the form of standardized questionnaires [2]. These are qualitative measures of subjects’ perceptions of positive or negative impact of oral diseases and their treatment on their everyday life established, for example, with the oral health-related quality of life questionnaire (OHRQoL) [3]. Measurement is nowadays considered as an essential element in oral health investigations, especially to determine the repercussions of oral diseases or abnormalities and related treatments on the daily lives of individuals [4]. A large number of measurement instruments have been developed and validated for schoolchildren and adolescents to obtain clinical data on the impact of oral health on their quality of life. They include the Child Perceptions Questionnaire (CPQ) [5–7], the Child Oral Impacts on Daily Performances Index (C-OIDP) [8], the Child Oral Health Impact Profile (COHIP) [9], and the Pediatric Oral Health-Related Quality of Life (POQOL) [10]. Investigations of the repercussions of oral health in young children are lacking due to the absence of paediatric indices to evaluate theses impacts. Furthermore, young children often have cognitive limitations in revealing past events due to their immaturity. However, oral health-related quality of life can be assessed validly and reliably both in self-reports from children as young as 36 months of age and by asking parents/guardians about their perceptions of their child’s OHRQoL [11].

Few instruments are available to evaluate the OHRQOL of children aged 0 to 5 years old: the Michigan Oral Health Related Quality of Life scale-child version (MOHRQoL) [11], the Scale of Oral Health Outcomes for 5-year-old children (SOHO-5) [12] the Early Child Oral Health Impact Scale (ECOHIS) [13]. MOHRQoL and ECOHIS were designed only for parental-reporting. ECOHIS is a well-known instrument for assessing oral health quality of life in children aged 0–5 years old and their families and has been translated into several languages, adapted to different cultures and validated in several countries including France, China, Iran (Farsi), Brazil, India (Urdu) and many others [14–24] but not yet In Madagascar. Such work allows comparing oral health impacts and treatment needs in children at the international level using standardized instruments, and also takes into account their cultural effects. Children under 5 years of age may have many oral health problems, such as teething pains, Early Childhood Caries (ECC) and dental trauma. The ECC is major oral health problem in disadvantaged populations, affects preschool children worldwide and significantly impacts on children’s oral health-related quality of life. In Madagascar, 85% of 6 year-old children have untreated dental caries in primary teeth [25]. There is currently no instrument available in Madagascar to measure the impacts of oral health status on the quality of life of pre-school age children and their families. For this issue, the ECOHIS was chosen in this study. The ECOHIS questionnaire is a parent-assessed OHRQoL. It has been shown capable of capturing the impact of a variety of oral conditions on the quality of life of preschool children and families in several countries. It is acknowledged that before using a measurement instrument in a country, it must be translated and be subjected to cultural adaptation and psychometric property evaluation. The objectives of this cross-cultural study were to translate, validate and analyse the psychometric properties of the Malagasy version of the ECOHIS on children aged 3 to 5 years old.

## Methods

A cross-sectional study was performed in Mahajanga city to test the psychometric characteristics of the Malagasy version of the ECOHIS questionnaire.

The target population was the parents or other adults accompanying young children. The young children were recruited randomly in two public kinder gardens. To be included in the study, the children had to be aged between 3 to 5 years old, and be accompanied by a Malagasy-speaking parent/caregiver. For the parents/caregiver to be included, they had to be living with the child and be able to speak, read and write Malagasy. The eligible parents (*N* = 150) were informed verbally regarding the objectives of the study and verbal consent was requested. Parents had to self-complete the ECOHIS questionnaire and additional questions on their level of education were asked, The Ethics Committee of the Ministry of Health gave its agreement for the study under number 34-MSANP/CE, on 17 March 2015.

Access to the original questionnaire was possible since it is in an article available through Open Access distributed according to the terms of the Creative Commons Attribute License (http://creativecommons.org/licenses/by/2.0) of Pahel et al. [13].

### Clinical examination

Before administering the questionnaire, a clinical examination of the preschool children was performed by two dentists familiar with caring for children. The dentists were trained to carry out clinical examinations; a calibration test was performed and the statistical Kappa coefficient was 0.87. The dental caries of primary teeth were recorded according to World Health Organisation (WHO) criteria for the visual evaluation of tooth decay in classrooms, by using mean counts of decayed, missing (due to caries) and filled teeth (dmft) [26]. The PUFA index was used in the clinical examination to record the severity of the consequences of untreated dental decay. The PUFA index records the presence of visible pulpal involvement (p); ulceration caused by dislocated tooth fragments (u), fistula (f), and abscess (a) (PUFA) [27] reported as mean score. The PUFA score per person is calculated cumulatively as DMFT. The children were divided into two groups: group 1(PUFA free) and group 2 with PUFA ≥1.

#### Cultural and language adaptation

##### Translation of the ECOHIS questionnaire into Malagasy

In order to use the questionnaire for a population with a different language and socio-cultural context, the original version had to be translated and adapted cross-culturally. The transcultural adaptation was carried out according to international recommendations [28, 29]. The procedure for evaluating the psychometric properties of a questionnaire consisted of two main steps: a translation phase followed by a phase of reliability and validity evaluation.

The translation phase was structured in 5 steps: forward translation, synthesis by an evaluation committee, back translation, comparison and a pre-test phase.

For the forward translation, two persons, (a dentist familiar with caring for young children and a linguist) translated the original ECOHIS questionnaire into Malagasy independently of each other.

### Synthesis

Once the translations were received, an evaluation committee composed of the two translators, the senior investigator and the authors met and compared the translations from the linguistic, conceptual, practical and cultural angles to finally agree on the 1st version of ECOHIS in Malagasy.

### Back translation

An English teacher and a certified translator were asked to back translate this 1st Malagasy version into English. These translators were unaware of the existence of the original version.

### Comparison

Once the back-translations were ready, the committee compared them with the original version of ECOHIS in order to discuss possible divergences, evaluate the quality of the translation and obtain a preliminary final version of the questionnaire in Malagasy.

### Pre-test and content validity

This penultimate version was pre-tested for face validity by interviewing the parents (or accompanying adults) of young children aged 3 to 5 years old. The translated questionnaire was self-completed by a sample of 15 parents who spoke, read, and wrote Malagasy perfectly. The pre-test terminated with an open discussion between the respondents and the two interviewers. All the comments expressed by the respondents (questions, words, or sentences that they felt were difficult to understand) were noted and revised by external experts to obtain a final consensual version of the questionnaire. Thus, for instance, instead of the literal translation of the word guilty it was finally decided to use a semantic equivalence: *feeling of responsibility* or *tompon’andraikitra* in Malagasy. The pre-test permitted rectifying the imperfections of the first draft of the translated questionnaire. It evaluated the clarity of the questionnaire, the understanding of the words and how the sentences were written. The accuracy items were evaluated during the committee’s meetings. The members of the experts committee pursued a discussion approach until consensual agreement was obtained. The final Malagasy version of the ECOHIS questionnaire was then ready for cross-cultural evaluation.

#### Measure

The ECOHIS is an English language questionnaire. It was developed in the United States to assess the impact of oral health problems and related treatments on the quality of life of preschool children aged 3 to 5 and their families [13]. The scale comprises 13 items, and has two main sections: the Child Impact Section (9 items) and the Family Impact Section (4 items). The *Child Impact Section* has four domains: *child symptom* (one item), *child function* (four items), *child psychology* (two items) and *child self-image and social interaction* (two items). The *Family Impact Scale* has two domains: *Parental distress* (two items*)* and *Family function* (two items). The response options are coded: 0 = *never*; 1 = *hardly ever*; 2 = *occasionally*; 3 = *often*; 4 = *very often.* And *I don’t know* response option was also proposed in the American version. Two global rating questions were added at the end of the ECOHIS questionnaire.

### Score calculation

ECOHIS scores were calculated simply as the sum of response codes for the child and family sections separately. The values could range from 0 to 52 for the total scale (0–36 for the child section and 0–16 for the family section). The highest score expressed a higher impact of the state of oral health whereas the lowest score expressed a lower impact. For the domains, the scores were ordered as follows: 0 to 4 for the *Child Symptom domain*; 0 to 16 for the *Function domain* (4 items); and 0 to 8 for the last two domains, *Psychology* and *Self-image*. In the Family Impact Section there are two domains containing two items each: *Parental distress* and *Famil*y *function* and the scores for each domain range from 0 to 8.

#### Evaluation of the reliability and validity of the Malagasy version of ECOHIS

Test-retest reliability was evaluated using an intraclass correlation coefficient (ICC*)* calculated by a two-way analysis of variance [30]. Thirty parents (2 infants/parents per item was applied, i.e. a minimum of 26 respondents were expected) responded to the items and 2 weeks later they completed the scale a second time. The children were chosen for the stability of their oral health conditions. A period of 2 weeks to 1 month was generally accepted between the test and the retest [31, 32]. An ICC value lower than 0.7 was considered as doubtful reliability; acceptable between 0.7 and 0.8; good between 0.8 and 0.9; and excellent from 0.9 upwards [30].

Internal consistency was measured by calculating Cronbach’s alpha for the child and family sections separately, and for the entire scale. A Cronbach’s alpha coefficient higher than 0.70 was considered as reliable [33].

Construct validity is the extent to which an instrument measures the construct that it is intended to measure. Construct validity was achieved when the questionnaire:

1. measured the differences between contrasting groups of participants,
2. reflected the framework hypothesized in a hypothesis testing study, and
3. could undergo a confirmatory factor analysis that adequately establishes that the measurement model fitted the actual data [34].

Thus, the validation of the construct was evaluated by analysing the convergent validity, the factor structure and the discriminant validity.

To determine the convergent validity of the Malagasy-ECOHIS questionnaire, the parents were asked to answer the two global health-rating questions added at the end of the scale. These subjective self-reported health measures were:

- *In general, how would you rate the dental health of your child?*
- *In general, how would you rate the overall health of your child?*

The responses options for these questions were scored from 1 to 5 as: 1 = *excellen*t; 2 = *very good*; 3 = *good*; 4 = *fair* and 5 = *poor*. Convergent validity was evaluated based on Spearman’s rank order correlation between the scale scores, the scores of the child and family sections and the two global rating questions. Determining the convergent validity consisted in examining the hypothesis, which assumed that parents who declared a high level of impact on the scale would report a poorer state of oral health for the children than parents who declared a low level of impact.

Construct validity was also evaluated by performing exploratory factor analysis using both the principal component analysis with Varimax rotation and Maximum Likelihood with oblique rotation (direct oblimin). Sample adequacy and the appropriateness of the factor analysis were evaluated previously by the Kaiser-Meyer-Olkin (KMO) test and Bartlett’s sphericity test. Exploratory Factor Analysis was conducted to extract the new factor structure and examine the construct validity. The number of extracted components was determined by scree plot and the percentage of variance explained by each component. A factor was considered important if its eigenvalue exceeded 1.0 and the significance level was set at *p* < 0.05 (two sided) [35]. Model fit was analysed in order to investigate whether the structure of the factors could be reproduced in the Malagasy ECOHIS dataset.

To evaluate the construct validity and confirm the single dimension of the Malagasy version of ECOHIS, a partial confirmatory factor analysis (PCFA) was performed on the 13 items after the exploratory factor analysis. Several model fit indices were used to examine the goodness-of-fit of the model, including the Normed Fit Index (NFI), Comparative Fit Index (CFI), Tucker Lewis Index (TLI) [36] and Root Mean Squared Error of Approximation (RMSEA) [37]. An ideal score of CFI, an NFI > 0.70, a TLI > 0.90, and RMSEA scores < 0.10 were indicative of an acceptable model [38].

An ideal score of CFI, NFI was > 0.70, TLI was > 0.90, and RMSEA scores < 0.10 were indicative of a good model [39].

Regarding discriminant validity, the hypothesis was that the Malagasy version of ECOHIS would be capable of distinguishing pufa-free young children from those with pufa ≥1. Since caries-free preschool children are scarce in the study population, the group who presented pufa = 0 were compared with the group with a pufa ≥1. Therefore, the parents of children belonging to the pufa ≥1 group should have a higher ECOHIS score (indicating a poorer oral health-related quality of life) than preschool children with no major oral health problem. The differences were assessed using the Mann Whitney U test. To estimate the effect size, statistics were calculated by dividing the mean ECOHIS scores by the standard deviation (SD). The effect size was evaluated by calculating the difference between the means of the ECOHIS total scores for the group with pufa = 0 and that with pufa> 1 and dividing the difference by the standard deviation (SD) of the summary scores of the group with pufa = 0. Effect size statistics of < 0.2 indicate a small, clinically meaningful magnitude of difference; effect size statistics of 0.2–0.7 indicate a moderate difference, and effect size statistics of > 0.7 indicate a large difference [40].

##### Criterion validity

Ideally, criterion validity would be measured relative to a gold standard. As no such standard existed for oral health related quality of life measures, and as the criterion had to be a widely accepted measure, a pain item was used for assessing criterion validity. Criterion validity was assessed by comparing the correlation between the total ECOHIS score, subscale scores and the variable pain obtained from the first item of the child impact section, using Spearman’s correlation coefficient. Criterion validity was checked by a correlation coefficient. Correlation coefficients equal to 0.70 or over were desirable [41].

Data processing and the statistical analyses were performed using the software Statistical Package for Social Sciences (SPSS) 24.0. The significance levels of the statistical tests were set at 0.05. According to the authors’ recommendations, all the respondents giving the response “*I don’t know*” *(DK)* to more than 2 items in the parent section and more than 1 item in the infant section should be excluded from the analysis.

## Results

### Cultural adaptation and content validity

The original number of items and domains for the two sections (child impact section and family impact section) were preserved in the Malagasy version. The committee of experts confirmed the relevance of the translated version.

Table 1 presents the socio-demographic characteristics of the children and respondents (*N* = 150). The children were aged from 3 to 5 years old. 78.0% of the respondents were mothers among whom, 56.7% had not finished their secondary school, and only 9 had been at high school. The mean (Standard Deviation) DMFT index of the preschool children was 6.01(1.71) Clinical groups were: children with PUFA = 0 (*n* = 81) and children group with PUFA ≥1 (*n* = 69).

**Table 1:** Demographic characteristic of preschool children and parents respectively

Demographic characteristic	Frequency		Mean (SD)
	n	%	
Child’s characteristics			
Age			
3	40	26.7	4.17(0.82)
4	44	29.3	
5	66	44.0	
Gender			
Boys	65	43.3	
Girls	85	56.7	
Parent’s characteristics			
Relationship to child			
Mother	117	78.0	
Father	33	22.0	
Level of education			
Unfinished secondary school	88	56.7	
Secondary school	56	37.3	
High school	6	6.0	
Oral health status (dmft^a^)			6.01(1.71)
Clinical groups			
Children with			
pufa = 0	81	54.00	
Children with			
pufa≥1	69	46.00	

^a^decayed, missing, filled teeth = dmft; pulp involvement (p), ulceration (u), fistula (f) and abscess (a) = pufa

The Table 2 shows that in this study sample, *pain* (66.0%), *difficulty eating* (54.0%), *trouble sleeping* (48.7%), *frustration* (48.0%) and *missing school* (46.7%) were the most frequently reported in the *Child Impact Section* whereas in the *Family Section*, the impact of *financia*l (63.3%) and *felt guilty* (58.0%), were the most frequently reported. In this evaluation, the Impact on the family was higher (58.2%) than the impact on the child (51.6%). Four *“Don’t Know” (DK)* answers were noted in four separate items. No respondent had more than one *DK* answer. In the Child Impact Section three parents choose *DK* answer on the items related to *pain*, to *drinking hot and cold beverage* and to *pronouncing word.* In the *Family Impact Section*, one parent gave *DK* response option on the item *felt guilty.*

**Table 2:** Distribution of the Malagasy ECOHIS responses (n = 150)

Impacts	Never/ hardly ever n(%)	Occasionally/ often /or very often n(%)	Don’t know n(%)
Impact on the Child			
Child symptom			
1. How often has your child had **PAIN** in the teeth, mouth or jaws	50(33.3)	99(66.0)	1(0.7)
Child function			
How often has your child … … because of dental problems or dental treatments?			
2. Had difficulty **DRINKING** hot or cold beverages	89(59.3)	60(40.0)	1(0.7)
3. Had difficulty **EATING** some foods	69(46.0)	81(54.0)	0(0.0)
4. Had difficulty **PRONOUNCING** any words	84(56.0)	65(43.3)	1(0.7)
5. Had missed preschool, day care or school (**ABSENCE**)	80(53.3)	70(46.7)	0(0.0)
Child psychology			
6. Had trouble **SLEEPING**	77(51.3)	73(48.7)	0(0.0)
7. Been irritable or **FRUSTRATED**	78(52.0)	72(48.0)	0(0.0)
Child self-image and social interaction			
8. Avoided **SMILING** or **LAUGHTHING**	86(57.3)	64(42.7)	0(0.0)
9. Avoided **TALKING**	110(73.3)	40(26.7)	0(0.0)
Impact on Family			
Parental distress			
How often have you or another family member …. because of dental problems or dental treatments?			
10. Been **WORRIED/UPSET**	68(45.3)	82(54.7)	0(0.0)
11. Felt **GUILTY**	62(41.3)	87(58.0)	1(0.7)
Family function			
12. Taken time off from **WORK**	65(43.3)	85(56.7)	0(0.0)
13. How often has your child had dental problems or dental treatment that had a financial impact on your family **FINANCIAL**	55(36.7)	95(63.3)	0(0.0)

#### Reliability

Test-retest responses revealed an ICC of 0.889. For internal consistency, Cronbach’s alpha coefficients test values were 0.807 for the Child impact section, 0.822 for the Family impact section, and 0.847 for the overall Malagasy-ECOHIS score. When the items were deleted one by one, the values did not increase for any of the items. All values of Cronbach’s alpha exceeded 0.70. The coefficients correlation of corrected items, part of the internal consistency reliability assessment, ranged from *r* = 0.477 to *r* = 0.770 for child impact section. The lowest coefficients were related to *difficulty pronouncing words* for the child impact section and *felt guilty* for the family impact section and the highest values belonged to *difficulty eating* and *taken time off from work* for child impact section and family impact section respectively (Table 3).

**Table 3:** Cronbach’s alpha coefficient for child impact section and family impact section assessed separately

	Complete Correlation of corrected items	Cronbach’s alpha if item deleted
Child Impact Section		
1 Oral/dental pain	.669	.839
2.Difficulty in drinking	.653	.841
3.Difficulty in eating	.707	.839
4.Difficulty in pronouncing words	.595	.841
5.Missed pre-school/school	.655	.841
6.Difficulty in sleeping	.707	.840
7.Irritable or frustrated	.770	.837
8.Avoided smiling or laughing	.703	.840
9.Avoided talking	.722	.842
Family Impact Section		
10.Been upset	.618	.840
11.Felt guilty	.477	.843
12.Work	.637	.841
13.Financial	.614	.842
ECOHIS total scale	.847	

#### Construct validity

The correlation between the global ratings (of oral health and overall health) and the ECOHIS total score was found to be statistically, positively significant at *p* < 0.001 (*r* = 0.763 and *r* = 0.694, respectively). Table 4 also shows that the ECOHIS total scores and scores for both ECOHIS sections were significantly, positively related to the parents’ global ratings of their children’s oral health (*r* = 0.763; *r* = 0.781; *r* = 0.494 *p* < 0.001, respectively) and overall health (*r* = 0. 694; *r* = 0.760 and *r* = 0.378 *p* < 0.001, respectively).

**Table 4:** Criterion validity

ECOHIS	Pain	
	*r*	*p*
Total scale	.611	.000
Child impact section	.624	.000
Family impact section	.400	.000

*r*-Spearman correlation coefficient; *p* value

#### Factor analysis

The Bartlett’s test of sphericity was significant (where khi2 = 707.983 for a degree of freedom (df) =78 and p < 0.001). This indicated that the items of the Malagasy-ECOHIS version were correlated. A KMO index of 0.864 indicated the adequacy of the sampling and thus an exploratory factor analysis could be conducted.

The exploratory factor analysis extracted three main factors with Eigenvalues > 1 and combined; they were capable of explaining 65.21% of the variances of which the first factor taken alone explained 46.52%.

#### First factor

Child oral health, function and socio- environmental impact, comprised 7/9 items of the Child impact section (items, 1, 2, 3, 5, 6, 7, 9).

#### Second Factor

Family emotional and social impact grouped 3 /4 items of the Family impact section (10,11,12).

#### Third Factor

Social Well-being contained three items (items 4, 8, 13) (Table 5).

**Table 5:** Construct validity: rank correlations between total scale and subscales scores, and global ratings of oral health and overall health (*n* = 150)

ECOHIS	Global ratings of			
	Oral health		Overall health	
	*r*	*p*-value	*r*	*p*-value
Total scale	.763	0.000	.694	0.000
Child impact section	.781	0.000	.760	0.000
Subscales				
Symptom	.554	0.000	.527	0.000
Function	.650	0.000	.613	0.000
Psychology	.468	0.000	.510	0.000
Self-image	.467	0.000	.509	0.000
Family impact section	.494	0.000	.378	0.000
Parental distress	.445	0.000	.344	0.000
Family function	.502	0.000	.380	0.000

*r*-Spearman correlation coefficient; *p* value

#### Partial confirmatory factor analysis (PCFA)

The Maximum Likehood Estimation (MLA) associated with PCFA solution was equal to 80,874 with 42 degrees of freedom and a *p* value < 0.001 which was smaller than the corresponding null model khi2 of Bartlett’s test of sphericity. Based on these two khi2 values, the model-fit indices were calculated to be NFI = 0.88, TLI = 0.88, CFI = 0.93, and RMSEA = 0.07.

The Visual Summaries of Fit of distributions of the residuals showed that the frequency distributions of the correlation residuals or covariance residuals had a normal shape.

#### Discriminant validity

In the two sections of ECOHIS (child impact and family impact), the mean scores were significantly higher in the group of preschool children with mean PUFA count ≥1 compared to the group with PUFA = 0 (*p* < 0.001) (Table 6), The effect size was > 0.70 for the total scale scores, which indicated a moderate to large difference (child impact section).”

**Table 6:** Factor analysis

Item N°	Item	Factor 1	Factor 2	Factor 3
		Child oral health: function and socio- environmental impact	Family emotional and social impact	Social Well-being
1	Pain	**.745**		
2	Drinking hot/cold	**.679**		
3	Eating some foods	**.605**		
4	Difficulty pronouncing			.**799**
5	Missed preschool	**.773**		
6	Trouble sleeping	**.740**		
7	Irritable, frustrated	**.767**		
8	Avoid smiling			**.658**
9	Avoid talking	**.591**		
10	Been upset		**.702**	
11	Felt guilty		**.775**	
12	Taken time		**.691**	
13	Financial impact			**.596**
% variance explained (total = 65.216%)		46.524	10.789	7.903

Varimax rotation with Kaiser normalisation; extraction method: analysis of principal components

#### Criterion validity

The relationships between ECOHIS total score and subscales (Child impact and Family impact), and *pain* as criterion measure were significantly correlated, with Spearman’s rank correlation coefficients of *r* = 0.61; *r* = 0.62; *r* = 0.40 respectively (Table 7).

**Table 7:** Discriminant validity: overall and subscale scores for parents of children pufa free and children with pufa≥1

ECOHIS	Clinical groups					
	Pufa = 0		Pufa≥1		*p*-value^*^	Effect size
	Mean (SD)	Median	Mean (SD)	Median		
Total scale	13.53(8.71)	12	20.88(9.63)	21	0.000	0.84
Child impact section	7.86(5.83)	6	13.77(6.00)	15	0.000	1.01
Family impact section	5.67(3.84)	6	7.12(3.71)	8	0.020	0.37

Standard Deviation (SD) ^*^Mann Whitney test

## Discussion

The aim of this study was to evaluate the psychometric properties of the Malagasy version of the ECOHIS questionnaire by examining its content, internal consistency, test-retest reliability, convergent, construct, discriminant validity, and criterion validity.

Few instruments have been developed to assess the quality of life for preschool children below the age of 6 years old. ECOHIS is one of the most frequently used questionnaires used to determine such quality of life. The difference between existing measures was that ECOHIS information on the OHRQoL of preschool children was obtained only through parental reports. This was the main raison why we chose the ECOHIS questionnaire. It was important that the parents’ perceptions of the impact of oral and dental conditions on the child’s and family’s daily life were assessed. The resulting scale was obtained from the first OHRQoL questionnaire for preschool children evaluated for Madagascar. In the present study, the cross-cultural adaptation of the Malagasy version of ECOHIS was based on international recommendations [28, 29]. The result of the forward-backward translation process showed wordings similar to the original ECOHIS. During the translation phase, we pre-tested the questionnaire to identify misunderstandings of questions. Face and content validity were determined. The results demonstrated that all the items were understood and accepted by the respondents during the pre-test. This procedure was imperative for the cross-cultural and social adaptation of the tool for measuring oral health-related quality of life. For the main study, 150 parents were included. The sample size might be criticised for being small but according to Clark et al., a sample size between 100 to 200 can be considered acceptable [42]. In general, a ratio of 5–10 subjects to 1 item has also been recommended [43]. In addition, according to the quality criteria required to measure the characteristics of the questionnaire on health suggested by Terwee et al., at least 50 subjects were necessary to evaluate the construct validity and the reproducibility analysis, and at least 100 subjects were required to analyse internal consistency [40]. The distribution of the responses to all the items showed that in our study, the highest mean scores were obtained in the domains of symptom (pain) and function (eating difficulties, sleeping disorders, irritation or frustration, and missing school). They were the impacts most frequently reported by the parents. These findings were consistent with the studies conducted in France, Brazil, and Lithuania, and with the original questionnaire [13, 14, 18, 20]. However, the strong impact of the item “finance” for the family impact section appeared specific to this study. This financial difficulty could explain the high prevalence of decayed teeth remaining untreated reported in the results. The impact of socio economical status on the oral health related quality of life was largely reported in the literature. The authors found that a low socio economical status (SES) could negatively affect the oral health related quality of life of children [44]. In the present study, reliability assessed by both the test-retest and internal consistency was satisfactory. Values were higher than 0.70, in line with what was demonstrated by all the previous versions [13, 14, 17, 18]. The test-retest score of 0.88 was consistent with the findings of the original study of ECOHIS (0.84), and the Farsi (0.82), Urdu (0.80) and Chinese (0.64) versions [13, 15, 18, 24]. These results showed a good level of agreement between the test and the retest data and reflected the good reliability of the Malagasy ECOHIS version. In order to evaluate the Construct validity, the global ratings of children’s overall health and oral health status perceived by parents were correlated with the ECOHIS total scale and subscales. The results showed that global ratings of oral health and overall health were significantly and positively correlated to the ECOHIS scale and subscales. Similar findings were reported by the other studies where parents who perceived worse oral health status had significantly higher ECOHIS scores [13–15, 17, 19].

Moreover, the factor analysis model suggested three-factor models with adequate goodness-of-fit indicators. The exploratory factor analysis revealed three-factor structures instead of two sections with six domains, as in the original version [13] To discuss the model fit of CFA, it has been suggested that a RMSEA value less than 0.08 is good [45]. Therefore, the RMSEA value of 0.07 in this study sample indicated an acceptable fit. The NFI and TLI index values of 0.885 should both be over 0.90 for a good fit, but in this sample the two indices are below this threshold. The CFI of 0.938 was above 0.90, which showed a good fit. Based on these indices, the sample had an acceptable fit to the 3-factor model. The Malagasy version of ECOHIS was also able to discriminate between children who presented clinical consequences of untreated decayed teeth and those without consequences, measured by the pufa index. The original ECOHIS questionnaire was judged reliable and capable of distinguishing children with different levels of tooth decay [13]. To sum up, in the construct, convergent, concurrent and discriminant validity tests, the analysis showed that the validity of the Malagasy version of ECOHIS is acceptable. Overall tests used to assess the reliability and validity of ECOHIS showed acceptable performance. Although acceptable, the results of the factor analysis were liable to criticism since the sample had fewer than 300 subjects. Indeed, some authors recommended at least 300 subjects to perform a factor analysis [42]. Tabachnick & Fidell (2007) reported that a ratio of 10 participants per item is sufficient for an exploratory principal component/factor analysis [46]. Since ECOHIS has 13 items, a minimum sample size of 130, therefore, appears suitable to perform an exploratory factor analysis. For Guadagnoli and Velicer, a sample of 150 observations is enough to obtain an adequate solution in a factor analysis as long as the inter-correlations are reasonably strong [47].

### Limitations, directions for future research

Although this study demonstrated that the Malagasy version of ECOHIS obtained good reliability and validity results, other studies should be performed to confirm these characteristics on patients for use in clinical research. Furthermore, other investigations should be carried out to evaluate the reactivity of this version of the ECOHIS questionnaire. The sample was not representative of all preschool children in Madagascar, thus the results cannot be generalized. This Malagasy version of ECOHIS should be tested further on other different populations with different clinical diseases to determine its discriminant properties in other clinical situations. This cross-sectional study did not allow measuring the responsiveness of the instrument. A longitudinal study should be conducted in the future.

## Conclusion

Globally, this Malagasy version of the ECOHIS questionnaire can be considered to have good psychometric properties and could be used on the Malagasy parents of preschool children to measure the impact of oral health status on child and family quality of life.

## Data Availability

Yes.

## Abbreviations

CFI: Comparative Fit Index
COHIP: Child Oral Health Impact Profile
C-OIDP: Child Oral Impacts on Daily Performances Index
CPQ: Child Perceptions Questionnaire
DMFT index: Decayed, missing (due to caries) and filled teeth
ECC: Early Childhood Caries
ECOHIS: Early Child Oral Health Impact Scale
ICC: Intraclass Correlation coefficient
MOHRQoL: Michigan Oral Health Related Quality of Life
NFI: Normed Fit Index
OHRQoL: Oral Health-Related Quality of Life questionnaire
PCFA: Partial Confirmatory Factor Analysis
POQOL: Paediatric Oral Health-Related Quality of Life
PUFA index: Pulpal involvement (p); ulceration (u), fistula (f) and abscess (a)
RMSEA: Root Mean Squared Error of Approximation
SOHO-5: Scale of Oral Health Outcomes for 5-year-old children
SPSS: Statistical Package for Social Sciences
TLI: Tucker Lewis Index
WHO: World Health Organisation
